# Redox and metal profiles in human coronary endothelial and smooth muscle cells under hyperoxia, physiological normoxia and hypoxia: Effects of NRF2 signaling on intracellular zinc

**DOI:** 10.1016/j.redox.2023.102712

**Published:** 2023-04-23

**Authors:** Matthew J. Smith, Fan Yang, Alexander Griffiths, Alexander Morrell, Sarah J. Chapple, Richard C.M. Siow, Theodora Stewart, Wolfgang Maret, Giovanni E. Mann

**Affiliations:** a King's British Heart Foundation Centre of Research Excellence, School of Cardiovascular and Metabolic Medicine & Sciences, Faculty of Life Sciences & Medicine, King's College London, 150 Stamford Street, London, SE1 9NH, UK; b London Metallomics Facility, Faculty of Life Sciences & Medicine, King's College London, UK; c Research Management & Innovation Directorate (RMID), King's College London, UK; d Departments of Biochemistry and Nutritional Sciences, School of Life Course & Population Sciences, Faculty of Life Sciences & Medicine, King's College London, UK

**Keywords:** Human coronary artery, Coronary artery endothelial cells, Coronary artery smooth muscle cells, Metals, Metallomics, LA-ICP-MS, ICP-MS, Zn, ZnT1, HIF-1α, NRF2, HO-1, NQO1, Metallothionein, Redox status, Physiological normoxia, Hyperoxia, Hypoxia, Oxygen

## Abstract

Zinc is an important component of cellular antioxidant defenses and dysregulation of zinc homeostasis is a risk factor for coronary heart disease and ischemia/reperfusion injury. Intracellular homeostasis of metals, such as zinc, iron and calcium are interrelated with cellular responses to oxidative stress. Most cells experience significantly lower oxygen levels *in vivo* (2–10 kPa O_2_) compared to standard *in vitro* cell culture (18kPa O_2_). We report the first evidence that total intracellular zinc content decreases significantly in human coronary artery endothelial cells (HCAEC), but not in human coronary artery smooth muscle cells (HCASMC), after lowering of O_2_ levels from hyperoxia (18 kPa O_2_) to physiological normoxia (5 kPa O_2_) and hypoxia (1 kPa O_2_). This was paralleled by O_2_-dependent differences in redox phenotype based on measurements of glutathione, ATP and NRF2-targeted protein expression in HCAEC and HCASMC. NRF2-induced NQO1 expression was attenuated in both HCAEC and HCASMC under 5 kPa O_2_ compared to 18 kPa O_2_. Expression of the zinc efflux transporter ZnT1 increased in HCAEC under 5 kPa O_2_, whilst expression of the zinc-binding protein metallothionine (MT) decreased as O_2_ levels were lowered from 18 to 1 kPa O_2_. Negligible changes in ZnT1 and MT expression were observed in HCASMC. Silencing NRF2 transcription reduced total intracellular zinc under 18 kPa O_2_ in HCAEC with negligible changes in HCASMC, whilst NRF2 activation or overexpression increased zinc content in HCAEC, but not HCASMC, under 5 kPa O_2_. This study has identified cell type specific changes in the redox phenotype and metal profile in human coronary artery cells under physiological O_2_ levels. Our findings provide novel insights into the effect of NRF2 signaling on Zn content and may inform targeted therapies for cardiovascular diseases.

## Highlights

• Physiological O2 (5kPa O) alter redox phenotype of coronary endothelial -(HCAEC) but not smooth muscle (HCASMC).cells
• Inductively coupled plasma mass spectrometry establishes that O_2_ differentially affects metal content in HCAEC and HCASMC
• siNRF2 reduces total intracellular Zn under 18 kPa O_2_ in HCAEC but not HCASMC.
• NRF overexpression increases in intracellular Zn in HCAEC, but not HCASMC, under 5 kPa O_2_.

## Graphical abstract

**Figure:**
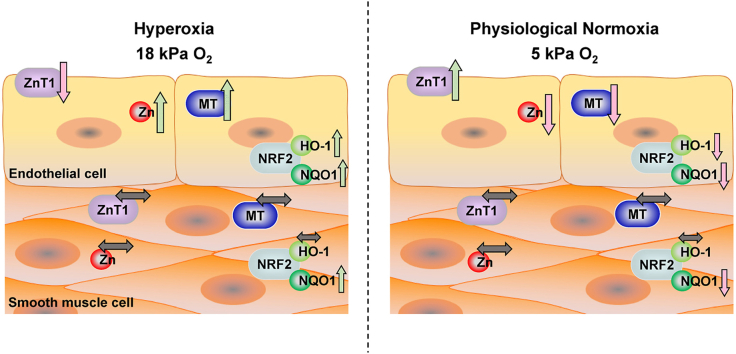

## 1 Introduction

Coronary artery and heart disease are leading causes of mortality worldwide, yet only recent studies have focused on the association between these conditions and trace metal micronutrients such as zinc (Zn) [[1], [2], [3], [4], [5], [6]]. Dysregulation of Zn^2+^ homeostasis is associated with ischemia/reperfusion injury [4,5,[7], [8], [9]]. Serum zinc levels are low in patients undergoing cardiac surgery and, although an association between zinc deficiency and postoperative outcomes remains to be established [10], elevated zinc levels and zinc supplementation are associated with reduced post MI scarring [11] and cardiomyopathy [12].

Although zinc is a redox-inert metal, physiological concentrations of zinc have antioxidant, anti-inflammatory and anti-proliferative properties [[13], [14], [15], [16], [17], [18]], whilst zinc deficiency or overload generates oxidative stress [19]. Intracellular zinc concentrations change dynamically to regulate both rapid cellular events and slow transcriptional responses [3,[20], [21], [22]]. In human cells, the concentration of free Zn^2^ ions r estimated at a few hundred picomolar but the total zinc concentration approximates one hundred micromolar [3]. Free Zn^2+^ can increase transiently above low nanomolar concentrations, yet its role as a signaling ion is tightly regulated by cytosolic buffering and the activity of transporters that remove Zn^2+^ ions from the cytosol [20,22,23]. ZnT (solute-linked carrier 30) transport proteins lower zinc concentrations through cellular efflux or uptake into cellular compartments, whilst ZIP (solute-linked carrier 39) proteins increase zinc levels through influx into cells or export from organelles [24,25]. In addition, metallothioneins (MT), small proteins containing thiolate clusters, bind Zn^2+^ ions with a range of affinities [19,26]. Thus, tight control of cellular Zn^2+^ levels via multiple transporters maintains cellular function, including thiol/disulfide redox homeostasis because the majority of intracellular zinc is bound to redox-sensitive thiols of the amino acid cysteine [3,23]. Increases in free Zn^2+^ alter gene transcription through their modification of critical proteins controlling cellular signaling.

Endothelial and smooth muscle take up Zn^2+^ [27], and zinc protects the endothelium against oxidative stress [28,29]. Sustained increases in intracellular Zn^2+^ in human endothelial cells leads to apoptotic cell death as a consequence of impaired glutathione homeostasis and mitochondrial ATP synthesis [30], further highlighting the intricate cross-talk between zinc, reactive oxygen species and endogenous antioxidant defenses regulated by nuclear factor (erythroid-derived 2)-like 2 (NRF2) [[31], [32], [33], [34], [35]]. In contrast, zinc deficiency induces an inflammatory endothelial cell phenotype, associated with increased monocyte adhesion which can be reversed by zinc supplementation [36]. Moreover, zinc supplementation has been shown to restore autophagic flux in heart failure patients [12].

The importance of physiologically relevant O_2_ levels in cell culture *in vitro* has been reviewed [[37], [38], [39], [40], [41], [42]]. Compared to cells cultured in standard incubators under atmospheric oxygen (∼18 kPa O_2_), most cells experience significantly lower O_2_ levels *in vivo* [[37], [38], [39], [40]], with the blood-dissolved O_2_ gradient in coronary arteries ∼5 kPa. We previously reported that long-term (5 days) adaptation of endothelial cells to physiological O_2_ (5 kPa) increases the bioavailability of nitric oxide [43,44] and activity of sarco/endoplasmic reticulum Ca^2+^ATPase (SERCA), protecting cells against ionomycin-induced Ca^2+^ overload [45]. Furthermore, we have shown that NRF2-regulated redox signaling is attenuated in umbilical vein and brain microvascular endothelial cells under physiological normoxia [46,47]. The present study correlates redox and metal profiles in human coronary artery endothelial cells (HCAEC) and smooth muscle cells (HCASMC) under hyperoxia (18 kPa O_2_), physiological normoxia (5 kPa O_2_) and hypoxia (1 kPa O_2_), focusing on the effects of NRF2 on intracellular zinc. To our knowledge, our findings provide the first insight into cell type specific regulation of intracellular metals in coronary artery endothelial and smooth muscle cells adapted to different pericellular O_2_ levels.

## 2 Methods and materials

### 2.1 Long-term culture of HCAEC and HCASMC under defined O_2_ levels

Primary human coronary artery endothelial cells (HCAEC) and smooth muscle cells (HCASMC) were obtained from PromoCell (Germany). HCAEC were cultured in Endothelial Cell Growth medium MV2 (PromoCell), supplemented with growth medium MV2 supplement pack (PromoCell) and 1% penicillin (100 U/ml)/streptomycin (100 μg/ml). HCASMC were cultured in Smooth Muscle Cell Basal Medium 2 (PromoCell), supplemented with growth medium 2 supplement pack and 1% penicillin (100 U/ml)/streptomycin (100 μg/ml). HCAEC and HCASMC monolayers were maintained for at least 5 days in O_2_-controlled Scitive Dual or InVivo500 workstations (Baker, USA), gassed to 18, 5 or 1 kPa O_2_ under 5% CO_2_ at 37 °C. All cell culture treatments and experiments were conducted with cells within the O_2_-controlled workstations and/or an O_2_-controlled plate reader (CLARIOstar, BMG Labtech, Germany) to avoid re-exposure of cells to room air and stabilization of HIF-1α [43,44,46,47].

### 2.2 Intracellular O_2_ levels in HCAEC and HCASMC measured using MitoXpress®-INTRA

Intracellular O_2_ levels in live cells were monitored using MitoXpress®-INTRA (Agilent Technologies, USA), which is a cell-penetrating phosphorescent platinum-porphyrin based nanoparticle probe [46,47]. HCAEC and HCASMC were seeded into black clear bottomed 96-well plates and loaded with the nanoparticle probe (10 μg/ml) for 16 h in complete medium. Phosphorescence signals at 655 ± 55 nm after excitation at 355 ± 55 nm were measured after 30 μs (D1) and 70 μs (D2) with a 30 μs window and converted to probe lifetime (T) [T=(D2–D1)/ln (l_w1_/l_w2_)], where T represents emission lifetime and l_w1_ and l_w2_ represent signals measured at window 1 and window 2. Averaged lifetime values measured at 7 ambient O_2_ tensions (18, 15, 10, 7.5, 5, 2.5, 1 kPa) were plotted against the known O_2_ tension and subjected to an exponential fit analysis. Lifetime values were then converted to O_2_ (kPa) by a curve based on the parameters of the fit. Lifetime values were then interpolated from this curve to give the dissolved intracellular O_2_ level in HCAEC and HCASMC cytosol (see Fig. 2C and D). Dissolved O_2_ in culture medium was also measured in parallel by diluting MitoXpress®-INTRA (2.5 μg/ml) in complete medium.

### 2.3 Oxygen consumption rate in HCAEC and HCASMC

Cells were adapted for 5 days to 18 or 5 kPa O_2_ in an O_2_-controlled workstation and seeded into Thermo Nunc flat bottom 96-well microplates in standard culture medium for 48 h. After cells reached ∼80% confluence under 18 or 5 kPa O_2_, the medium was changed and a Resipher oxygen sensing lid (Lucid Scientific Inc., USA) was attached [48]. Basal oxygen consumption rate (OCR) was measured over 10 h in cells under 18 or 5 kPa O_2_. Data were analyzed using the Resipher web application (Lucid Scientific) and OCR values expressed as fmol/mm^2^/s/μg protein (see Supplementary Fig. 1).

### 2.4 Metallomic profiling in cells adapted to 18, 5 or 1 kPa O_2_ using ICP-MS and LA-ICP-MS

HCAEC and HCASMC lysates were collected in purified trace metal free water with a resistivity ≥18.2 MΩcm obtained from a Milli-Q system (Merck Millipore, USA). Lysis solutions were subjected to 3 freeze-thaw cycles and sonication before heating with nitric acid 65% Suprapur® (Merck Millipore, USA) to 95 °C for 2 h. Subsequently, the lysis solution was cooled and diluted with purified ddH_2_O to a final concentration of 0.5% nitric acid (0.1 M). Quantification of total zinc (Zn), copper (Cu), manganese (Mn) and magnesium (Mg) concentrations in HCAEC and HCASMC lysates was conducted using a PerkinElmer NexION 350D Inductively Coupled Plasma Quadrupole Mass Spectrometer (ICP-QMS) under Dynamic Reaction Cell (DRC) and Kinetic Energy Discrimination (KED) modes [49]. Cell lysate samples were introduced to the ICP-QMS via a Cetac ASX-520 autosampler (Teledyne, Cetac, USA) coupled to a SeaSpray glass nebulizer fitted to a quartz cyclonic spray chamber. Measurements in counts per second were converted to a concentration by applying a regression model from calibration standards (multi-element standard solution VI, Sigma-Aldrich). Concentrations were then normalised to the amount of cells in the lysis solution based on the protein concentration.

Laser ablation inductively coupled plasma mass spectrometry (LA-ICP-MS) was used to map Zn distribution in HCAEC and HCASMC cultured on 8-well glass removable chamber slides (Ibidi, Germany). For the LA-ICP-MS experiments, an Analyte Excite 193 nm ArF*excimer-based laser ablation system (Teledyne Photon Machines, USA) equipped with a HelEx II two-volume ablation cell was coupled to an iCAP TQ ICP-MS (Thermo Fisher Scientific, USA) via a Aerosol Rapid Introduction System (ARIS). Elemental images of cell monolayers were acquired under fixed dosage mode (10 laser shots per pixel) with a laser energy density of 0.8 j cm^−2^ and a beam waist diameter of 2 μm (effective image resolution). ICP-MS measurement acquisition parameters were optimised based on cell monolayer washout times, which provided a dwell time of 36.8 ms for Zn under KED mode. Total cell content of metals was calculated using ImageJ software (National Institute of Health, USA).

### 2.5 Measurement of intracellular ATP and glutathione levels

HCAEC and HCASMC were adapted to 18, 5 or 1 kPa O_2_ for 5 d and ATP and glutathione (GSH) extracted using 6.5% trichloroacetic acid (Sigma-Aldrich, UK). To measure ATP, cell extracts were incubated with firefly lantern extract (Sigma, UK) containing both luciferase and luciferin, while GSH levels were determined using a fluorometric assay [46,47,50]. Luminescence and fluorescence were measured in a plate reader (CLARIOstar, BMG Labtech, Germany) and expressed as nmol/mg protein.

### 2.6 Immunoblotting

Whole cell lysates were collected using SDS lysis buffer supplemented with protease inhibitor cocktail (Sigma-Aldrich, UK). Denatured samples were separated by gel electrophoresis, electro-transferred onto polyvinylidene difluoride membranes (Millipore, Sigma, USA) and then probed with primary and HRP-conjugated secondary antibodies (Millipore, Sigma, USA). Membranes were probed for HIF-1α (Abcam, UK), catalase (Calbiochem, UK), CuZnSOD (Abcam, UK), MnSOD (R&D Systems, Minneapolis), NQO1 (Santa Cruz, USA), HO-1 (BD Biosciences, USA), Bach1 (Santa Cruz, USA), metallothionein (MT1/2, Abcam, UK), ZnT1 (Abcam, UK) and β-actin (Sigma-Aldrich, UK). Membranes were subjected to development with enhanced chemiluminescence (Millipore, Sigma, USA) with images captured using a G:Box system (Syngene, UK). Immunoblot densitometry data were analyzed using ImageJ software (National Institute of Health, USA).

### 2.7 siRNA Nrf2 silencing

HCAEC and HCASMC were adapted to 18 or 5 kPa O_2_ for 5 d and then transfected with 15 pmol/well and 8.84 pmol/well respectively with either scrambled siRNA or Nrf2 siRNA (Santa Cruz, USA) for 24 h, using FuGENE (Promega, UK) or Dharmafect 1 transfection reagent (Thermo Scientific, UK) according to the manufacturer's instructions [47].

### 2.8 Nrf2 overexpression

HCAEC and HCASMC were adapted to 18 or 5 kPa O_2_ for 5 d in 24-well plates and then transfected with control vector pcDNA3.1 (3.1C) or pcDNA3.1-hNrf2 (hNrf2) vector at 100 ng per well for 24 h, using FuGENE transfection reagent (Promega, UK) according to the manufacturer's instructions.

### 2.9 Statistical analysis

Data denote the mean ± S.E.M. of 3–6 different HCAEC or HCASMC cultures and were analyzed using Graphpad Prism 9. Significance was assessed using either an unpaired Student's *t*-test or one- or two-way ANOVA followed by a Bonferroni Post Hoc test where appropriate, with *P < 0.05, **P < 0.01, ***P < 0.001 and ****P < 0.0001 considered significant.

## 3 Results

### 3.1 Intracellular O_2_ levels in HCAEC and HCASMC during culture under physiological normoxia

**Fig. 1:**
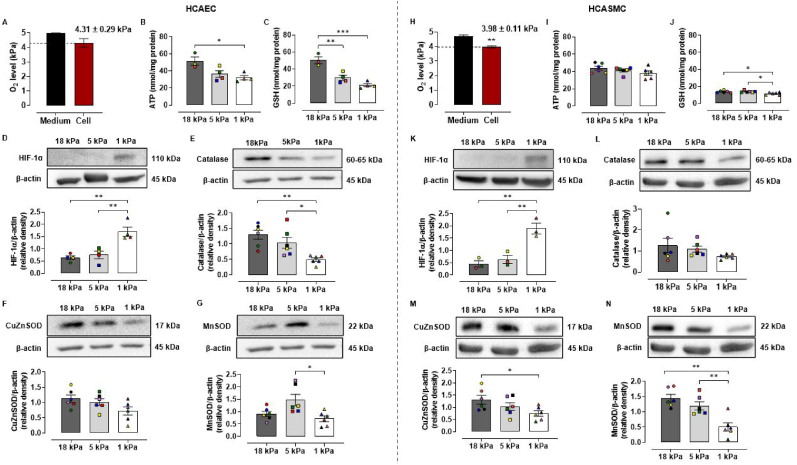
O_2_ dependent changes in redox phenotype of HCAEC and HCASMC adapted to 18, 5 or 1 kPa O_2_ **A and H**, O_2_ content in the cytosol (red column, dashed line) and culture medium (black column) of HCAEC and HCASMC adapted for 5 d to 5 kPa O_2_ using MitoXpress®-INTRA. **B–C and I-J**, Intracellular ATP and total GSH levels in HCAEC and HCASMC adapted for 5 d to 18, 5 or 1 kPa O_2_. **D-G and K–N**, representative immunoblots and densitometric analysis of HIF-1α, catalase, CuZnSOD and MnSOD expression relative to β-actin under 18, 5 or 1 kPa O_2_. Data denote mean ± S.E.M., n = 3–6 independent cell cultures (color coded), unpaired Student's *t*-test or one-way ANOVA followed by a Bonferroni Post Hoc test analysis, *P < 0.05, **P < 0.01, ***P < 0.001. (For interpretation of the references to color in this figure legend, the reader is referred to the Web version of this article.)

We previously reported that NRF2 regulated antioxidant defense enzymes are upregulated during culture of human umbilical vein (HUVEC) and murine brain microvascular (bEnd.3) endothelial cells under 18 compared to 5 kPa O_2_ [46,47]. As cellular responses to changes in pericellular O_2_ levels are cell type specific (reviewed in Ref. [37]), we conducted real-time measurements of intracellular O_2_ in HCAEC and HCASMC monolayers using the phosphorescent nanoparticle probe MitoXpress®-INTRA. Intracellular oxygen content was measured during stepwise reductions in ambient O_2_ (18–1 kPa) in an O_2_-controlled, time-resolved fluorescent plate reader and calculated from phosphorescent lifetime measurements [46,47]. Intracellular O_2_ levels of 4.31 ± 0.29 kPa and 3.98 ± 0.11 kPa were measured respectively in HCAEC and HCASMC cultured under 5 kPa O_2_ (Fig. 1A and H), recapitulating physiological O_2_ levels measured in human coronary vasculature *in vivo* [37].

**Fig. 2:**
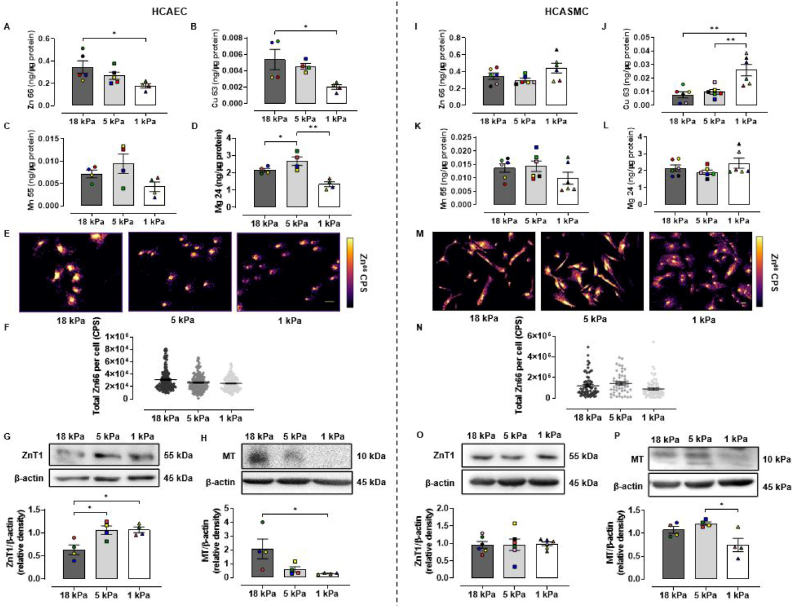
Metal homeostasis in HCAEC and HCASMC adapted to 18, 5 or 1 kPa O_2_ **A-D and I-L** Total Zn, Cu, Mn and Mg levels in HCAEC and HCASMC adapted for 5 d to 18, 5 or 1 kPa O_2_ measured by ICP-MS. **E and M**, spatial distribution of ^66^Zn counts per second (CPS) in HCAEC and HCASMC after 5 d culture under 18, 5 or 1 kPa O_2_ measured by LA-ICP-MS. **G-H and O–P**, representative immunoblots and densitometric analysis of ZnT1 and metallothionine (MT) expression relative to β-actin under 18, 5 or 1 kPa O_2_. Data denote mean ± S.E.M., n = 4–6 independent cell cultures (color coded), one-way ANOVA followed by a Bonferroni Post Hoc test analysis, *P < 0.05, **P < 0.01. (For interpretation of the references to color in this figure legend, the reader is referred to the Web version of this article.)

The basal oxygen consumption rate (OCR) was similar in HCAEC and HCASMC adapted to 18 kPa O_2_ (2.36 ± 0.08 *vs* 3.11 ± 0.05 fmol/mm^2^/s/μg protein) or 5 kPa O_2_ (1.09 ± 0.05 *vs* 1.16 ± 0.03, fmol/mm^2^/s/μg protein), with OCR values markedly lower in both cell types during long-term (5 d) culture under 5 kPa O_2_ (Supplementary Fig. 1).

### 3.2 Effects of O_2_ on HIF-1α stabilization and intracellular ATP and GSH in HCAEC and HCASMC

To determine whether 5 d culture under 5 or 1 kPa O_2_ induces a hypoxic response in HCAEC and HCASMC, HIF-1α stabilization was examined by immunoblotting. As shown in Fig. 1D and K, increased HIF-1α expression was only detected in cells adapted to 1 kPa O_2_, confirming the absence of an hypoxic phenotype in cells cultured under 5 kPa O_2_. We next examined whether changes in pericellular O_2_ levels affect intracellular ATP and total glutathione (GSH) levels. In HCAEC, ATP levels were only decreased under 1 kPa O_2_ (Fig. 1B), whereas ATP levels in HCASMC were affected negligibly (Fig. 1I). Intracellular GSH levels were significantly lower in HCAEC adapted to 5 and 1 kPa compared to 18 kPa O_2_ (Fig. 1C), consistent with our findings in bEnd.3 brain endothelial cells and airway epithelial cells [47,51]. Notably, basal GSH levels in HCASMC cultured under 18 kPa O_2_ (13.76 ± 0.58 nmol/mg protein) were significantly lower than levels in HCAEC (50.36 ± 4.11 nmol/mg protein, Fig. 1C) and reduced further during culture under 1 kPa O_2_ (Fig. 1J).

### 3.3 Oxygen dependent changes in antioxidant enzyme expression in HCAEC and HCASMC

Our previous studies indicated that cells cultured under physiological O_2_ levels are under lower oxidative stress and therefore express decreased levels of antioxidant enzymes [46,47]. Many of these enzymes such as CuZnSOD (SOD1) and MnSOD (SOD2) have metal constituents that are key for their function with cellular levels potentially regulated by oxygen. We therefore examined expression of catalase, responsible for the decomposition of H_2_O_2_, and SOD1 and SOD2, responsible for dismutation of superoxide. Catalase expression in HCAEC was decreased under both 5 and 1 kPa O_2_ compared to 18 kPa O_2_ (Fig. 1E), whilst CuZnSOD expression was affected negligibly (Fig. 1F). MnSOD expression was significantly lower in HCAEC under 1 kPa O_2_ compared to 5 kPa O_2_ (Fig. 1G). By comparison, CuZnSOD and MnSOD expression decreased in HCASMC under 1 kPa O_2_ compared to 18 kPa O_2_ (Fig. 1M and N). Taken together these data indicate a reduction in antioxidant enzyme expression in cells adapted to lower redox stress.

### 3.4 Adaptation to defined O_2_ levels alters intracellular metal content in a cell type specific manner

There is evidence that metal homeostasis, storage and channel expression are all affected by the oxygen environment and/or oxidative stress [[52], [53], [54], [55], [56]]. In particular, Zn has been reported to be protective against heart disease and ischemia-reperfusion. To determine whether metal homeostasis is influenced by the O_2_ environment, HCAEC and HCASMC monolayers were adapted to 18, 5 and 1 kPa O_2_ for 5 d and lysates analyzed by ICP-MS. In HCAEC, total Zn content decreased significantly between 18 and 1 kPa O_2_ (18 kPa = 0.345 ± 0.056 ng/μg protein, 5 kPa = 0.267 ± 0.032 ng/μg protein, 1 kPa = 0.177 ± 0.020 ng/μg protein) (Fig. 2A). However, no significant differences in Zn levels were detected in HCASMC under different O_2_ levels (18 kPa = 0.345 ± 0.090 ng/μg protein, 5 kPa = 0.298 ± 0.020 ng/μg protein, 1 kPa = 0.441 ± 0.058 ng/μg protein) (Fig. 2I). Interestingly, total Cu levels decreased significantly between 18 and 1 kPa O_2_ in HCAEC (Fig. 2B) but increased in HCASMC (Fig. 2J). There were no significant differences in total Mn content across the O_2_ conditions tested in either cell type (Fig, 2C and K). Under 5 kPa O_2_, HCAEC showed a significant increase in total Mg compared to both 18 and 1 kPa O_2_ (Fig. 2D), whilst HCASMC showed no difference (Fig. 2L). ICP-MS analysis of cell free culture media used for HCAEC and HCASMC revealed Zn concentrations of 2.0 μM and 2.8 μM, respectively (Table 1). Since the extracellular content of zinc in the culture media remained constant throughout experiments, our results indicate that changes in intracellular Zn levels are due to transport and not a passive reflection of the extracellular concentration.

**Table 1:** ICP-MS analysis of metal concentrations in HCAEC (MV2) and HCASMC (GM2) in cell-free culture media.

Culture medium	Zn	Cu	Mn	Mg
MV2 medium (HCAEC)	2.0 μM	114.6 nM	30.4 nM	1.0 mM
GM2 medium (HCASMC)	2.8 μM	85.9 nM	20.1 nM	4.5 mM

We then employed LA-ICP-MS to map the spatial distribution of Zn in HCAEC (Fig. 2E) and HCASMC (Fig. 2M). LA-ICP-MS analysis showed that ^66^Zn per cell trended to decrease in HCAEC as pericellular O_2_ levels were lowered (Fig. 2F), supporting our ICP-MS data (Fig. 2A). HCAEC demonstrated a concentrated distribution of zinc at the nucleus (Fig. 2 E). Whilst HCASMC also demonstrated a high nuclear localizaion, there was a stronger cytoplasmic signal compared to HCAEC at all O_2_ levels (Fig. 2M), with analysis of ^66^Zn per cell indicating a trend for a decrease in Zn content at 1 kPa O_2_ (Fig. 2N). The implications of these findings is that pericellular O_2_ levels may regulate the intracellular content of metals in a cell type specific manner, even in vascular cells isolated from the human coronary artery.

### 3.5 Zinc transporter ZnT1 and zinc-binding protein metallothionein (MT) expression correlate with intracellular zinc

Due to the importance of zinc in cardiovascular disease, we further interrogated the effects of O_2_ on Zn homeostasis by investigating the expression of some of the key proteins involved in zinc storage and transport. ZnT1 is the main zinc channel on the cell membrane responsible for zinc efflux [57]. Notably, ZnT1 protein expression was significantly lower in HCAEC adapted to 18 kPa O_2_ compared to 5 and 1 kPa O_2_ (Fig. 2G). Unlike HCAEC, ZnT1 expression in HCASMC was affected negligibly by changes in pericellular O_2_ levels (Fig. 2O). Metallothioneins are a family of cysteine-rich metal-binding proteins that bind a large proportion of the intracellular zinc pool [58,59]. Metallothionein (MT1/2) expression decreased as O_2_ levels decreased and was significantly lower in HCAEC adapted to 1 kPa O_2_ compared to 18 kPa O_2_ (Fig. 2H). This suggests that whilst the intracellular zinc-binding pool (MT) is increased at 18 kPa O_2_, cellular efflux capacity may be reduced, resulting in an increased total intracellular zinc. However, this does not hold true for HCASMC, where changes in O_2_ had no effect on total Zn content (Fig. 2N), although MT expression decreased significantly in cells adapted to 1 compared to 5 kPa O_2_ (Fig. 2P).

### 3.6 NRF2 knockdown reduces intracellular zinc in HCAEC but not HCASMC

**Fig. 3:**
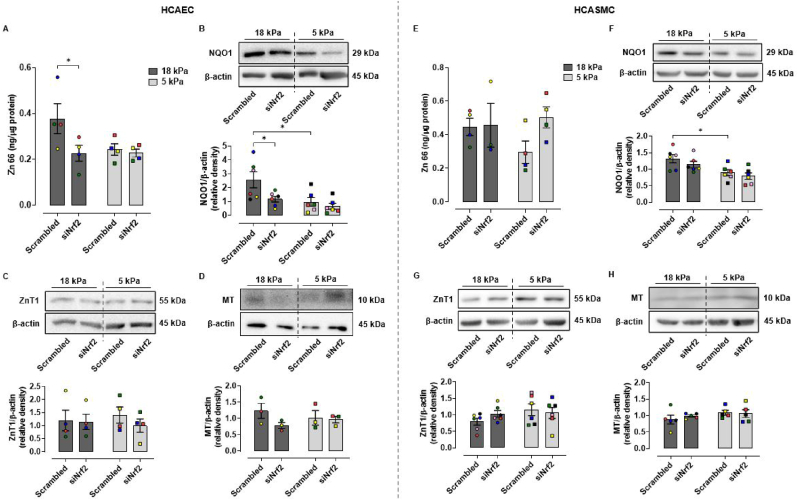
Effect of NRF2 silencing on Zn content and NQO1, ZnT1 and MT expression in HCAEC and HCASMC adapted to 18 or 5 kPa O_2_ A and D, Total Zn levels in HCAEC and HCASMC adapted for 5d to 18 or 5kPa O_2_ were measured by ICP-MS after transfection with scrambled or NRF2 siRNA for 24 h. **B–DandF-H**, representative immunoblots and densitometric analysis of NQO1, ZnT1 and MT expression relative to β-actin after silencing NRF2 transcriptional activity in cells adapted to 18 or 5 kPa O_2_. Data denote mean ± S.E.M., n = 4–6 independent cell cultures (color coded), two-way ANOVA followed by a Bonferroni Post Hoc test analysis, *P < 0.05. (For interpretation of the references to color in this figure legend, the reader is referred to the Web version of this article.)

As pericellular O_2_ levels affect NRF2 signaling [46,47] and NRF2 can modulate zinc homeostasis [[31], [32], [33]], we next investigated whether intracellular Zn levels were affected by NRF2 silencing in coronary artery cells adapted for 5 d under 18 or 5 kPa O_2_. In HCAEC under 18 kPa O_2_, Nrf2-siRNA resulted in a significant decrease in total intracellular Zn (scramble: 0.377 ± 0.065 ng/μg protein, Nrf2-siRNA: 0.227 ± 0.035 ng/μg protein, P < 0.05) as measured by ICP-MS (Fig. 3A), with Zn content similar to levels in HCAEC cultured under 5 kPa O_2_. Notably, there were no differences in Zn content between scramble and NRF2-siRNA transfected HCAEC under 5 kPa O_2_ (Fig. 3A). In contrast to HCAEC, NRF2-siRNA in HCASMC had negligible effects on total Zn content under any oxygen condition tested (Fig. 3E).

Consistent with our previous findings in other cell types [46,47], NQO1 expression was reduced in HCAEC and HCASMC transfected with scramble siRNA under 5 kPa O_2_ compared to 18 kPa O_2_ (Fig. 3B and F). In HCASMC, unlike HCAEC, NRF2-siRNA resulted in a modest reduction in NQO1 expression, although another NRF2 targeted HO-1 was significantly downregulated (data not shown). When we investigated whether changes Zn content correlated with changes in ZnT1 and metallothionein (MT) expression in HCAEC (Fig. 3C and D) and HCASMC (Fig. 3G and H), NRF2-siRNA had no effect on ZnT1 or MT expression.

### 3.7 Effects of NRF2 overexpression on total intracellular Zn and NQO1 expression

**Fig. 4:**
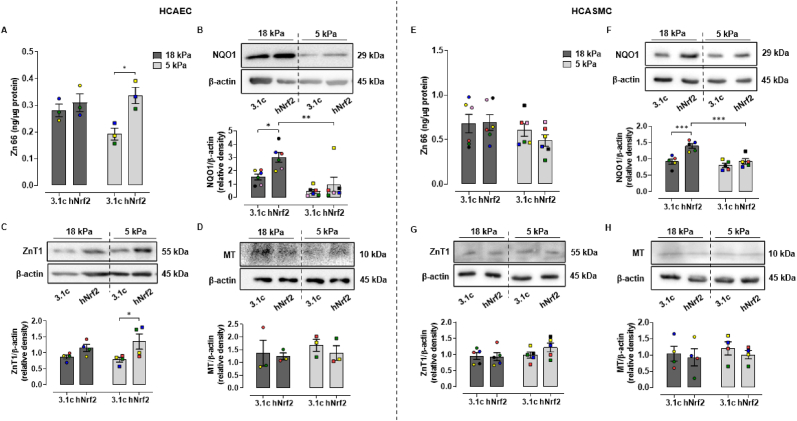
Effects of NRF2 overexpression on total Zn levels and NQO1, ZnT1 and metallothionein expression in HCAEC and HCASMC adapted to 18 kPa or 5 kPa O_2_ HCAEC and HCASMC were adapted for 5 d to 18 or 5 kPa O_2_ and then transfected with control vector pcDNA3.1 (3.1C) or NRF2 overexpression vector pcDNA3.1-hNrf2 (hNRF2) and cell lysates harvested after 24 h. **A and E**, Total Zn levels in HCAEC and HCASMC were measured by ICP-MS. **B–D and F–H**, Cell lysates were immunoblotted for NQO1, ZnT1 and MT expression relative to β-actin and analyzed by densitometry. Data denote mean ± S.E.M., n = 4–6 independent cell cultures (color coded), two-way ANOVA followed by a Bonferroni Post Hoc test analysis, *P < 0.05, **P < 0.01, ***P < 0.001. (For interpretation of the references to color in this figure legend, the reader is referred to the Web version of this article.)

Overexpression of NRF2 was achieved through transfection with a plasmid vector which constitutively expresses NRF2. Quantification of total Zn content in HCAEC by ICP-MS showed no differences in cells transfected with control vector (3.1C) or NRF2 expressing vector (hNRF2). However, overexpression of NRF2 at 5 kPa O_2_ tended to increase total Zn to levels in HCAEC under 18 kPa O_2_ (Fig. 4A). The NRF2 target NQO1 showed robust induction under 18 kPa O_2_, which was attenuated at 5 kPa O_2_ (Fig. 4B), consistent with our findings in HUVEC [46] and brain microvascular endothelial cells [47]. In HCAEC adapted to 5 kPa O_2_, overexpression of NRF2 increased ZnT1 expression (Fig. 4C) but had a negligible effect on MT expression (Fig. 4D). Total intracellular Zn in HCASMC was affected negligibly by NRF2 overexpression (Fig. 4E). In HCASMC, NQO1 was upregulated by NRF2 overexpression at 18 kPa O_2_ and attenuated at 5 kPa O_2_ (Fig. 4F), whilst ZnT1 and MT expression were affected negligibly (Fig. 4G and H).

### 3.8 Effects of sulforaphane on NRF2 activation and total Zn levels

We next investigated whether endogenous activation of NRF2 with sulforaphane (SFN, 2.5 μM) affected the expression of NQO1, ZnT1 or MT and total Zn levels in HCAEC and HCASMC. Despite robust induction of NQO1 in HCAEC, there was no significant effect on Zn content in either cell type under 18 or 5 kPa O_2_ (Supplementary Fig. 2). SFN increased expression of the zinc exporter ZnT1 in HCAEC under 5 kPa O_2_ but not under 18 kPa O_2_, with no significant effect on MT expression under either O_2_ condition. HCASMC showed no significant changes in Zn content, ZnT1 or MT following SFN treatment (Supplementary Figs. 2G and H). Although SFN did not induce NQO1 expression robustly in HCASMC, HO-1 expression was significantly upregulated in cells adapted to 18 or 5 kPa O_2_ (Supplementary Fig. 3B).

We previously reported that NRF2 activated HO-1 expression is attenuated in human endothelial cells under 5 kPa O_2_ due to an increase in expression of the repressor protein Bach1 [46]. When we probed Bach1 expression in HCAEC and HCASMC adapted to 18 or 5 kPa O_2_, basal and SFN induced Bach1 levels were only increased significantly in HCASMC under 5 kPa O_2_ (see Supplementary Fig. 4B).

## 4 Discussion

In view of the importance of zinc in influencing redox dysregulation in vascular [29], cardiac [9,38,57,60] and other cell types [33,61,62], our study in human coronary endothelial and smooth muscle cells cultured long-term under defined pericellular O_2_ levels has identified cell type specific differences in redox and metal profiles. In a recent expert recommendation, we emphasized the importance of recapitulating O_2_ levels in cell culture that reflect O_2_ levels *in vivo.* Adaptation of coronary endothelial and smooth muscle cells to 5 kPa O_2_ in our O_2_-controlled workstations enabled us to measure intracellular O_2_ levels that recapitulate measurements *in vivo*. Importantly, the absence of HIF-1α stabilization in both cell types under 5 kPa O_2_ confirms that cells were not exposed to hypoxia, consistant with our previous studies in other endothelial cell types [43,44,46,47].

Although intracellular ATP levels in HCAEC were affected negligibly when lowering O_2_ from 18 to 5 kPa, GSH content was significantly lower under 5 kPa O_2_, reflecting lower oxidative stress [47]. In HCASMC, lowering pericellular O_2_ from 18 to 5 kPa had negligible effects on GSH, antioxidant enzymes or NRF2 activated NQO1 expression. In contrast, GSH, catalase and NRF2 induced NQO1 expression were decreased in HCAEC under 5 kPa O_2_, highlighting differences in their redox phenotype and sensitivity to changes in pericellular O_2_. Moreover, the phenotype of HCAEC and HCASMC under 1 kPa O_2_ was characterized by lower intracellular GSH and antioxidant enzymes (e.g. catalase, SOD1 and SOD2), providing insights into the effects of long-term hypoxia on human coronary vascular cells. Despite enhanced expression of Bach1 in HCASMC under 5 kPa O_2_, these cells retained the ability to induce HO-1 expression most likey through non-NRF2 related transcription. In the context of cardiac function, inhibition of Bach1 may provide a novel target to protect the heart against pressure overload [63].

Endothelial cells synthesize ATP primarily via glycolysis with a low rate of O_2_ consumption [64]. Their relatively low mitochondrial content, low energy demand and high glycolytic activity enables endothelial cells to transfer more oxygen to perivascular cells (reviewed in Ref. [65]). Although the O_2_ demand of coronary endothelial cells is relatively low, glucose deprivation and hypoxia semstize these cells to changes in pericellular O_2_ levels [66]. In vascular smooth muscle cells, the rate of O_2_ consumption and lactate production are nearly equal on a molar basis under resting conditions, with ∼30% of the ATP supply from aerobic glycolysis and at least 90% of the flux via glycolysis converted to lactate [67]. Differences in metabolism may in part account for the differential sensitivity of HCAEC and HCASMC to changes in pericellular O_2_. Although basal oxygen consumption rate (OCR) was similar in HCAEC and HCASMC (see Supplementary Fig. 1), Sekine et al. reported marked differences in OCR between cardiomyocytes and other coronary cell types [68]. The high OCR in human iPSC cardiomyocytes was attributed to their large quantity of mitochondria and energy demand for spontaneous contraction [68]. In this context, a recent transcriptomic analysis of different human cancer cell lines cultured for 14 days under 18 *versus* 5 kPa O_2_ established that changes in O_2_ levels affect the expression of mitochondrial DNA-encoded genes in a highly cell type specific manner [69].

To our knowledge, the current study is the first to investigate the effects of controlled pericellular O_2_ levels on metal profiles in coronary artery endothelial and smooth muscle cells during long-term culture *in vitro*. Notably, total intracellular Cu content shows an opposite trend in HCAEC and HCASMC as O_2_ levels decreased from 18 to 1 kPa. The significant increase in Cu content in HCASMC under 1 kPa O_2_ may be associated with accelerated atherogenesis, as Heinecke et al. have shown that Cu supplementation in culture medium modifies low density lipoprotein (LDL) in human aortic smooth muscle cells, resulting in cholesteryl ester accumulation in macrophages [70]. Morevoer, HIF-1α mediated upregulation of mRNA/protein expression of Cu transposter 1 (CRT1) leads to increased Cu uptake in human pulmonary artery smooth muscle cells exposed to hypoxia, with elevated intracellular Cu enhancing proliferation and migration during hypoxia [71].

In recent years, an increasing number of studies have focused on the effects of hypoxia/reoxygenation or ischemia/reperfusion on intracellular Zn [4,72]. Notably, the majority of studies *in vitro* have compared the effects of hypoxia *versus* atmospheric O_2_, which is well known to enhance oxidative stress and reactive oxygen species generation in cultured cells [37]. Thus, we felt it important to measure total Zn content in coronary artery endothelial and smooth muscle cells adapted long-term to physiological normoxia (5 kPa O_2_) before examining the relationship between Zn and NRF2 regulated redox signaling. We have obtained the first comparative measurements of total Zn content in HCAEC and HCASMC under 18, 5 or 1 kPa O_2_ and established that O_2_ levels affect Zn content in HCAEC but not in HCASMC. This cell type specific sensitivity to pericellular O_2_ mediated changes in Zn content may reflect differences in the expression/activity of Zn transporters and/or binding proteins [73], but also the differing redox sensitivity of HCAEC and HCASMC to oxygen tension. In agreement, studies have shown that zinc modulation alters cardiac proteostasis [12].

To further invesitigate effects of NRF2 signaling on Zn content, HCAEC and HCASMC were transfected with NRF2-siRNA or NRF2 overexpression vector, or treated with the NRF2 inducer sulforaphane. Together our findings suggest that NRF2 activation/overexpression tends to increases total Zn content in HCAEC with negligible changes detected in HCASMC, consistent with the observed changes in ZnT1 and MT protein expression. In a previous study in HCAEC, downregulation of ZnT1 expression under depleted/low and high Zn conditions was attributed to a reduced turnover of new ZnT proteins [74], whilst in HUVEC low or oscillatory shear stress led to an upregulation ZnT1 and MT and lower intracellular free Zn [75]. As endothelial nitric oxide (NO) is decreased under oscillatory shear stress [76], Conway et al. suggested that reduced NO generation in atheroprone regions together with increased ZnT1 and MT expression could account for decreased intracellular free zinc [75].

The physiological concentration range of Zn is quite narrow and is strictly regulated by uptake, storage and secretion, mainly mediated by ZnT1-10 and ZIP1-14 and MT [21,77,78]. Notably, Tran et al. have shown that Zn transporters are located differentially among endothelial and smooth muscle cells in human subcutaneous microvessels [73]. ZIP10 and 14 are approximately equally expressed in both cell types, while ZIP1, 2, 8 and 12 are relatively more abundant in endothelial cells and ZIP14 is more abundant in smooth muscle cells [73]. Activation of NRF2 in HepG2 cells significantly increases mRNA levels of ZnT1, 3 and 6 and decreases ZnT10 and ZIP3 mRNA levels [31].

Transcriptional activation of metal regulatory transcription factor 1 (MTF-1) and NRF2 by oxidants is linked via a pool of available zinc controlled by MTF-1 [19]. Oxidation of Keap1 cysteine residues releases zinc, leading to nuclear accumulation of NRF2 and gene transcription, but it remains to be determined whether zinc release from Keap1 leads to transcriptional activation of MTF-1 [79]. Although in endothelial cells zinc activates NRF2 regulated glutamate-cysteine ligase and glutathione synthesis independent of MTF-1 [29], MTF-1 does play a role in detoxifying heavy metals and protection of cells against hypoxia and oxidative stress.

NRF2 and zinc influence the dynamic balance of cellular redox homeostasis, as evidenced in the present study in coronary vascular cells and in other cell types [[31], [32], [33]]. We have identified differences in the redox phenotype and metal profile in human coronary endothelial and smooth muscle cells adapted long-term to hyperoxia (standard cell culture), physiological normoxia (‘physioxia’) or hypoxia. Our findings further emphasize the importance assessing intracellular redox signaling and changes in total and labile zinc levels in cells under pericellular O_2_ levels encountered *in vivo*. To further recapitulate the physiological environment of coronary endothelial and smooth muscle cells, we encourage researchers not only to mimic O_2_ levels *in vivo* but to also investigate the effects of laminar shear stress and/or mechanical stress, noting that gene expression of metallothionein isoforms and ZnT1 are upregulated by low and oscillatory shear stress [75].

## Authors contributions

M.J.S., F.Y. and G.E.M. conceptualized the study; M.J.S., F·Y., A.G., A.M. developed the methodology; M.J.S., F.Y. performed and analyzed all experiments and A.G. and A.M. conducted the ICP-MS and LA-ICP-MS analyses; M.J.S., F.Y. and G.E.M. wrote the manuscript, which all authors reviewed. G.E.M. is the guarantor of this study, with responsibility for the integrity of the data and analysis.

## Declaration of competing interest

The authors declare that they have no conflicts of interest that could have influenced the study.

## Declaration of interest statement

Authors declare no conflicts of interest in this study.

## Data Availability

No data was used for the research described in the article.
