# Supplementary material for: Redox and metal profiles in human coronary endothelial and smooth muscle cells under hyperoxia, physiological normoxia and hypoxia: Effects of NRF2 signaling on intracellular zinc
Source: Redox Biol. 2023 Apr 23;62:102712. doi: 10.1016/j.redox.2023.102712 (PMC10165141; doi:10.1016/j.redox.2023.102712)
Supplement: Multimedia component 1 [file mmc1.docx]

**Redox Biology Smith et al. – Supplementary Data**

**Redox and metal profiles in human coronary artery endothelial and smooth muscle cells under hyperoxia, physiological normoxia and hypoxia: effects of NRF2 signaling on intracellular zinc**

Matthew J. Smith^1¶^, Fan Yang^1¶^, Alexander Griffiths^2^, Alexander Morrell^2^, Sarah J. Chapple^1^, Richard C.M. Siow^1^, Theodora Stewart^3^, Wolfgang Maret^4^ and Giovanni E. Mann^1*^

*^1^ King’s British Heart Foundation Centre of Research Excellence, School of Cardiovascular*

*and Metabolic Medicine & Sciences, Faculty of Life Sciences & Medicine, King’s College*

*London, 150 Stamford Street, London SE1 9NH, U.K.*

*^2^ London Metallomics Facility, Faculty of Life Sciences & Medicine, King’s College London*

*^3^ Research Management & Innovation Directorate (RMID), King’s College London*

*^4^ Departments of Biochemistry and Nutritional Sciences, School of Life Course & Population*

*Sciences, Faculty of Life Sciences & Medicine, King's College London*

^¶^ Authors contributed equally

* Corresponding author


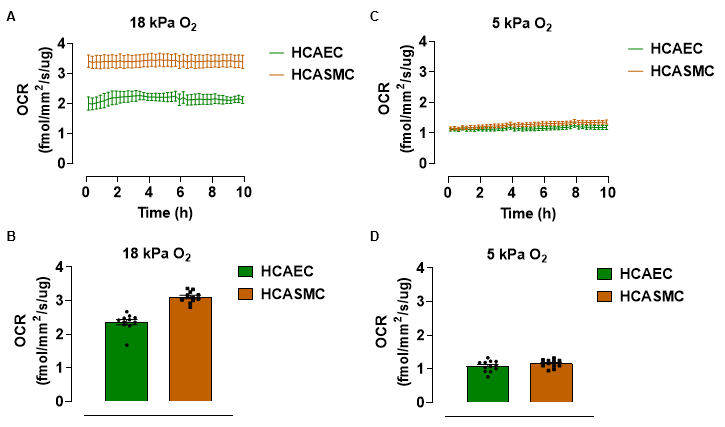


**Fig. S1 Basal oxygen consumption rate in HCAEC and HCASMC adapted to 18 or 5 kPa O_2_**

HCAEC and HCASMC were adapted for 5 d to 18 or 5 kPa O_2_ and then seeded into Thermo Nunc flat bottom 96-well microplates in standard culture medium for 48 h. Cells were maintained under 18 or 5 kPa O_2_ and, when monolayers reached ~80% confluence, the medium was changed and a Resipher oxygen sensing lid (Lucid Scientific, USA) attached. (**A and C**) Time course of basal oxygen consumption rate (OCR) in HCAEC and HCASMC measured over 10 h in the same 96-well plates under either 18 or 5 kPa O_2_ in an O_2_-controlled workstation. (**B and D**) OCR measurements normalized for cell protein and expressed as fmol/mm^2^/s/μg protein.


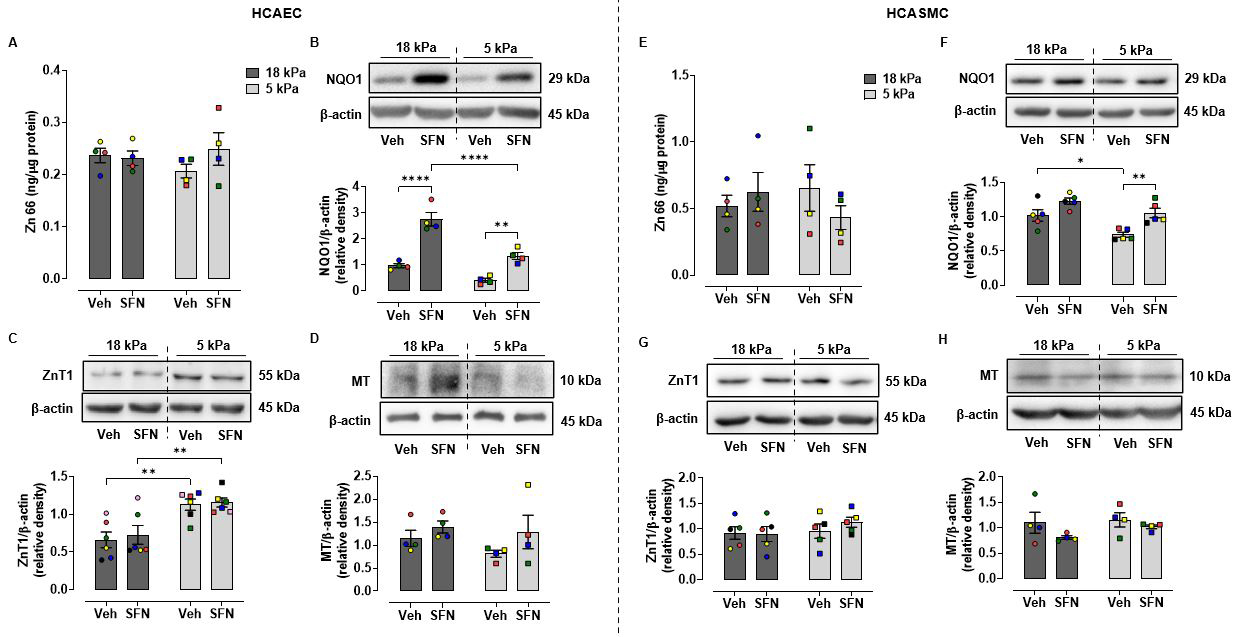


**Fig. S2 Effects of Nrf2 activation by sulforaphane on Zn content and expression of NQO1, ZnT1 and MT in HCAEC and HCASMC adapted to 18 kPa or 5 kPa O_2_**

**A and E**, Total Zn content in HCAEC and HCASMC adapted for 5 d to 18 or 5 kPa O_2_ measured using ICP-MS. Cells were treated with vehicle (0.01% DMSO) or the NRF2 inducer sulforaphane (SFN, 2.5μM) for 16 h, and cell lysates immunoblotted for NQO1, ZnT1 and MT expression relative to β-actin in HCAEC (**B-D**) and HCASMC (**F-H**) and analysed by densitometry. Data denote mean ± S.E.M., n = 4-6 independent cell cultures (each culture color coded), two-way ANOVA followed by a Bonferroni Post Hoc test analysis, **P*<0.05, ***P*<0.01, *****P*<0.0001.


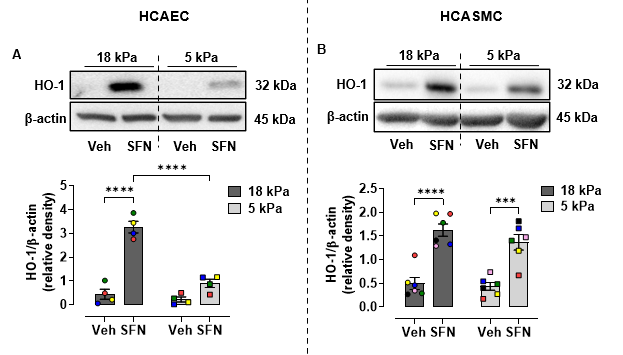


**Fig. S3 Sulforaphane induced HO-1 expression in HCAEC and HCASMC adapted to 18 or 5 kPa O_2_**

HCAEC and HCASMC were adapted for 5 d to 18 or 5 kPa O_2_ and then treated with vehicle (0.01% DMSO) or the NRF2 inducer sulforaphane (SFN, 2.5 μM 16 h). (**A and B**) Cell lysates were immunoblotted for HO-1 expression relative to β-actin and data analyzed by densitometry. Data denote mean ± S.E.M., n = 4-6 independent cell cultures (each culture color coded), two-way ANOVA followed by a Bonferroni Post Hoc test analysis, ***P*<0.01, ****P*<0.001. *****P*<0.0001.


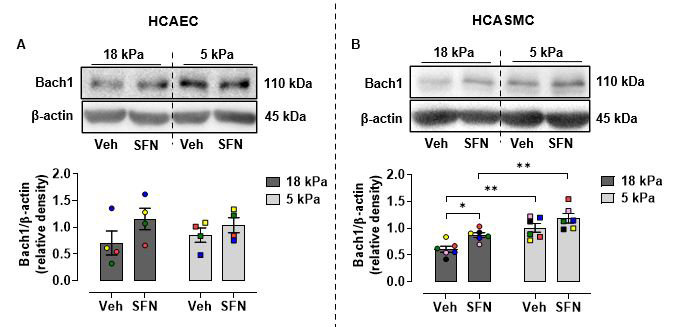


**Fig. S4 Basal and sulforaphane induced Bach1 expression in HCAEC and HCASMC adapted to 18 or 5 kPa O_2_**

HCAEC and HCASMC were adapted for 5 d to 18 or 5 kPa O_2_ and then treated with vehicle (0.01% DMSO) or sulforaphane (SFN, 2.5 μM) for 16 h. (**A and B**) Cell lysates were immunoblotted for Bach1 expression relative to β-actin and analyzed by densitometry. Data denote mean ± S.E.M., n = 4-6 independent cell cultures (each culture color coded), two-way ANOVA followed by a Bonferroni Post Hoc test analysis, **P*<0.05, ***P*<0.01.
